# Risk of COVID-19 Infection in Public Transportation: The Development of a Model

**DOI:** 10.3390/ijerph182312790

**Published:** 2021-12-04

**Authors:** Junsik Park, Gurjoong Kim

**Affiliations:** The Korea Transport Institute, Sejong-si 30147, Korea; gjkim0216@koti.re.kr

**Keywords:** COVID-19, infection risk, public transportation, distance measure, passenger reduction policy

## Abstract

South Korea’s social distancing policies on public transportation only involve mandatory wearing of masks and prohibition of food intake, similar to policies on other indoor spaces. This is not because public transportation is safe from coronavirus disease 2019 (COVID-19), but because no suitable policies based on accurate data have been implemented. To relieve fears regarding contracting COVID-19 infection through public transportation, the government should provide accurate information and take appropriate measures to lower the risk of COVID-19. This study aimed to develop a model for determining the risk of COVID-19 infection on public transportation considering exposure time, mask efficiency, ventilation rate, and distance. The risk of COVID-19 infection on public transportation was estimated, and the effectiveness of measures to reduce the risk was assessed. The correlation between the risk of infection and various factors was identified through sensitivity analysis of major factors. The analysis shows that, in addition to the general indoor space social distancing policy, ventilation system installation, passenger number reduction in a vehicle, and seat distribution strategies were effective. Based on these results, the government should provide accurate guidelines and implement appropriate policies.

## 1. Introduction

A novel coronavirus, the causative agent for coronavirus disease 2019 (COVID-19), was first identified in Wuhan City, Hubei Province, China, in December 2019, and it has since spread rapidly worldwide. The World Health Organization (WHO) declared it an international public health emergency in January 2020 and a global pandemic two months thereafter [1]. Since the first confirmed case at the beginning of 2020, the cumulative number of cases has exceeded 170,000 in South Korea as of July 2021 [2].

COVID-19 is a severe acute respiratory syndrome that is mainly transmitted through droplets from an infected person and can spread easily in daily life. The unprecedented rate of transmission and mortality of this infectious disease resulted in panic. Many countries and cities worldwide quarantined infected people to prevent the transmission of COVID-19 and implemented local lockdowns and facility closures, as well as movement restrictions. 

Unlike other countries, South Korea did not impose such measures. Instead, the policies focused on preventing the spread of the disease through early detection by expanding preemptive testing, isolation, and treatment of patients with confirmed COVID-19, tracking and managing close contacts; and limiting the size of gatherings. Residents were able to move freely with no restrictions; however, movement was still greatly reduced due to fear of infection. Many individuals started avoiding the use of public transportation and used personal vehicles instead.

In the Seoul metropolitan area, more than 15 million people use public transportation daily. Although the Korea Disease Control and Prevention Agency insisted that there were no confirmed cases of COVID-19 infection due to the use of public transportation, this seemed insufficient to relieve the fear. Transportation is crucial for daily life, and those without access to private vehicles have no choice but to rely on public means, despite the anxiety of contracting COVID-19. Because of the prolonged spread of COVID-19, the anxiety and precaution of people gradually faded, which led to the third and fourth waves of infection.

Experts now believe that the COVID-19 pandemic will not be resolved in the near future and that people will have to live with it as with the common cold. This calls into question the future of the use of public transportation, and whether the government needs to continue with these public transportation services without any clear countermeasures. The introduction of any policy regarding public transportation services should enable the prevention of COVID-19. However, the government needs to provide accurate information to relieve fear, while making efforts to reduce the spread of COVID-19. 

To date, many studies have been conducted on COVID-19 transmission to effectively contain its spread. Representative infection transmission models, such as the susceptible–infected–removed model [3,4,5,6,7] and data-driven time series [8,9], are macroscopic models. These models have the advantage of including macro-parameters and data. However, they cannot reflect the detailed factors affecting transmission, such as mask wearing, ventilation, and the environment. 

To address the limitations of these macroscopic models, several studies have been conducted to estimate the risk of infection [10,11]. The Rail Safety and Standards Board (RSSB) estimated the probability of COVID-19 transmission on rail transit and calculated the risk per journey by multiplying the ‘infection risk per passenger contact’ and ‘contacts per journey’ with the ‘impact of mitigating factors’, such as mask-wearing rate and ventilation rate. The probability of infection was estimated using three types of trains (44, 30, and 31 seaters), and the risk was 0.0045–0.0051%. They assumed the number of infected persons to be 0.0225, which is 0.05% of 45 passengers on a train. Lelieveld et al. estimated the risk of COVID-19 transmission in indoor environments using the infection risk of a single viral RNA copy and the density of the viral copies in the air considering exposure time, mask efficiency, and ventilation rate [11]. That study hypothesized that the virus spreads uniformly in one space, making it impossible to analyze the effect of the distance between people on the risk of COVID-19 transmission. 

Therefore, our study aimed to develop a model that can estimate the risk of COVID-19 infection and the number of infected individuals on public transportation, and to identify the influencing factors, including distance measures.

## 2. Materials and Methods

### 2.1. Disease Spread Model on Public Transportation: Assumption

#### 2.1.1. Route of Infection

The WHO estimates that the transmission routes of COVID-19 infection include contact, droplets, airborne, fomite, fecal–oral, bloodborne, mother-to-child, and animal-to-human transmission [12]. Among them, the transmission routes of infection on public transportation are possibly limited to droplets and airborne transmission. 

Respiratory tract secretions discharged during conversations or coughing are generally classified into droplets and aerosols depending on the particle size. The WHO defines droplets as particles > 5–10 μm in diameter and aerosols as particles of <5 μm [13]. Meanwhile, Hinds defined aerosols as particles of 0.001–100 μm in size that are suspended in a gas phase [14]. 

There is still a lack of consensus among experts on the standard for distinguishing between droplets and aerosols. Droplets are relatively large and cannot spread far under the influence of gravity, whereas aerosols are small and can spread farther. When aerosols float in the air and water evaporates, they form droplet nuclei that can penetrate the alveoli [15]. 

#### 2.1.2. Effectiveness of Masks

According to the SGS (Société Générale de Surveillance) [16], mask certification standards vary by country. The South Korean government is implementing a Korea Filter (KF) system for health mask certification, as shown in Table 1. A mask that has received KF80 certification means that it can block more than 80% of fine particles with an average particle size of 0.6 μm. Those certified as KF94 and KF99 can block 94% and >99% of particles, at an average particle size of 0.4 μm, respectively [17].

KF-certified masks have the advantage of lowering the risk of infection caused by external droplets due to their high dust-collection efficiency. However, this can result in breathing difficulty. Therefore, in June 2020, the Ministry of Food and Drug Safety added a Korea Filter Anti-Droplet (KF-AD) grade that prevents infection by blocking droplets and improves breathability. These masks are known to block 55–80% of fine particles with an average particle size of 0.4–0.6 μm.

Nevertheless, mask-wearing does not guarantee complete protection. Although the previously mentioned KF94 certified masks can block 94% of droplets and aerosols with a diameter > 0.4 μm, the remaining 6% can still penetrate. Moreover, since the inward leakage (IR) of KF94 is 11.0% or less, approximately 11% of air can leak through the gaps between the mask and the face. Thus, the possibility of infection caused by aerosol still exists. In addition, the risk of aerosol leakage can be higher with dental or cotton masks that are not KF certified.

Upon reviewing studies on the propagation of droplets and aerosols by mask type, Jayaweera et al. reported that dental masks allowed a significant leakage of 20–30% compared with the 5% allowed by elastomeric respirators and N95 masks certified in the US; they emphasized the importance of indoor social distancing, given that no mask can completely block the transmission of COVID-19 [18].

Although wearing a mask while using public transportation is the most basic means to reducing the spread of infection, there is still a risk of transmission, as mask-wearing does not completely block all droplets and aerosols. However, the risk of leakage may vary depending on the mask efficiency and the method of wearing it. 

### 2.2. 1-1 Infection Risk Model

Lelieveld et al. calculated the risk of COVID-19 transmission via indoor aerosols [11]. As shown in Equation (1), this risk was calculated using the infection probability of a single virus and the number of transferred viruses. In our study, the same model was used to calculate the risk of subject B being infected by the virus discharged by infected subject A.

(1) $$P\left(B\leftarrow A\right)=1-{\left(1-p\right)}^{n\left(B\leftarrow A\right)}$$

In this equation, *p* represents the risk of infection by a single virus, and *n(*B←A*)* represents the number of viruses discharged from infected person A to infect person B. 

**Figure 1:**
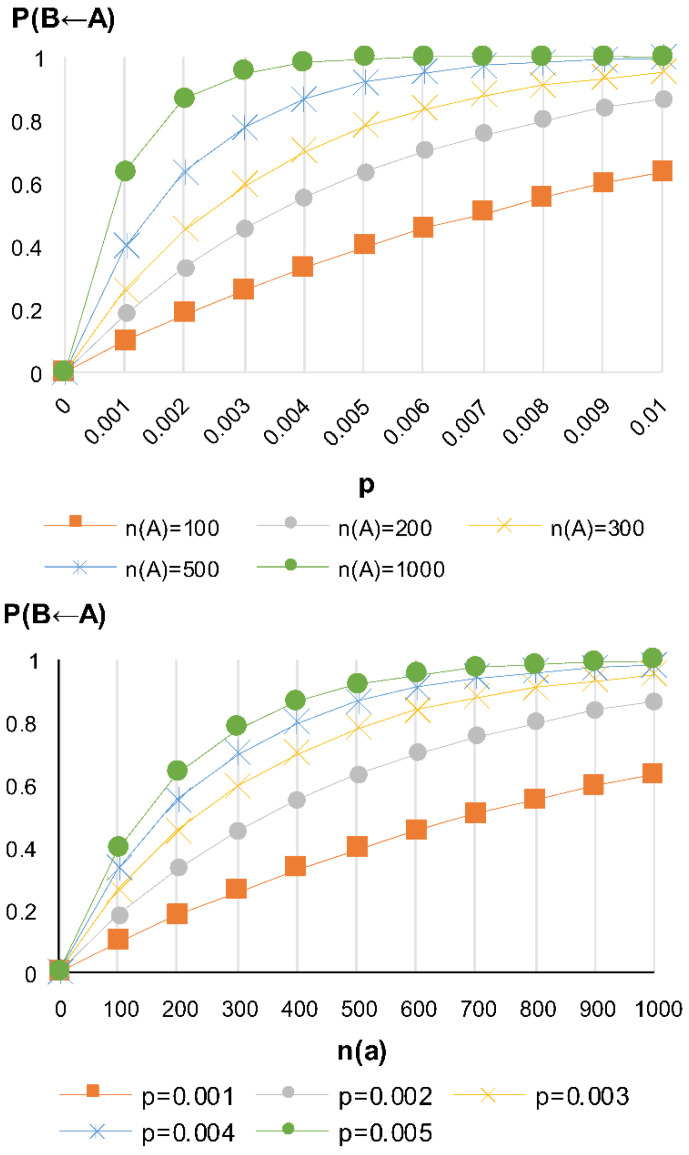
Risk of infection by a single virus in subject B according to the number of viral populations.

As shown in Figure 1, the risk of subject B being infected by infected person A increases with an increasing risk of infection by a single virus and an increasing number of viral populations that can cause infection. 

### 2.3. Virus Transmission Model

The number of viral populations transferred from person A to person B is unknown. In general, owing to the large particle size, the number of transmitted droplets is affected by gravity and decreases with an increasing distance. Nevertheless, aerosols, which are smaller and less affected by gravity, remain suspended in the air and travel farther. According to Wang et al., unlike other existing viruses, coronavirus is reported to spread through both droplets and aerosols [19]. 

Given that studies on the transmission pattern of individual coronaviruses are difficult to find in the literature, our study intends to present a distribution model based on two assumptions. The first assumption is that the risk of infection is the same for all people in the same space, as the virus floats in the air in the form of droplet nuclei and spreads uniformly regardless of the distance between infected and non-infected persons. The second assumption is that the risk of infection decreases with a decreasing number of viruses in the air and an increasing distance from the infected person, given that the density of the virus discharged from the infected person is lowered, while being spread by mixing with the surrounding air. 

#### 2.3.1. Uniform Distribution Model

In the model showing the risk of infection for subject B infected by infected person A, *n*(B←A) represents the number of viruses discharged from A to B. The number of transferred viruses is proportional to the number of viruses discharged by infected person A per hour and exposure time. The uniform distribution model assumes that the virus floats in the air in the form of a droplet nucleus and spreads uniformly in the same space. As the interior volume of the public transportation vehicle increases, the density of viral particles floating in the air decreases, exhibiting an inverse relationship, as shown in Equation (2):

(2) $$n1\left(B\leftarrow A\right)\;:\;f\left(h,\;w\right)\approx \left(\left(N\times h\right)\times \frac{b}{w}\right)\times R$$

where *N* denotes the number of aerosol particles discharged per unit time, *h* denotes the exposure time, *b* denotes the respiratory rate per hour ($\left[\frac{l}{h}\right])$, w denotes the interior volume of the vehicle ($\left[l=\frac{1}{1000}\times m^{3}\right])$, and *R* denotes the number of viruses per aerosol particle.

Since viruses are much smaller than aerosols in size, there are several individual viruses per aerosol particle. The number of viruses *R* per aerosol particle can be calculated as the product of the volume of aerosol particles v and the number of viruses per unit volume c.

The viruses discharged from infected person A are filtered to a certain extent by the mask of subject B, depending on the mask effectiveness, which had a value of 0–1. Higher effectiveness is demonstrated by a value closer to 1, and lower effectiveness by a value closer to 0. 

There are differences in the method and degree of ventilation depending on the operating characteristics of the public transportation mode. City buses and subways have a certain degree of ventilation achieved by the opening and closing of the doors at each station. At such times, a part of the aerosol discharged from infected person A moves outside the vehicle. Thus, the number of aerosol particles discharged from infected person A is reduced to a certain extent, depending on the level of ventilation. 

Moreover, since the virus discharged from infected person A must reach the respiratory tract of subject B to cause infection, the probability of virus deposition must be considered. Thus, when infected persons A and B stay together for h duration, the number of viruses that can be released by infected person A to cause infection in subject B can be summarized as shown in Equation (3):

(3) $$n1\left(B\leftarrow A\right)=\left(\frac{N}{f}\times h\right)\times \frac{b}{w}\times \left(c\times v\right)\times \left(1-m\right)\times t$$

where f denotes the ventilation rate ($\left[h^{-1}\right])$, m denotes the mask effectiveness, and *t* denotes the probability that the virus is deposited in the respiratory tract. 

The number of aerosol particles discharged by infected person A per unit time can be calculated from their respiratory rate. Thus, it can be calculated by differentiating between aerosol discharges when speaking and during respiration without conversation and then subtracting the amount of discharge filtered by the mask of infected person A. 

The number of aerosol particles *N* discharged by infected person A per unit time was calculated using Equation (4):

(4) $$N=(c_{b}\times \left(1-s\right)+c_{t}\times s)\times b\times \left(1-m\right)$$

where $c_{b}$ denotes the number of aerosol particles per unit volume discharged during respiration ($\left[\mathrm{Number}\;\mathrm{of}\;\mathrm{aerosol}\;\mathrm{particles}/{\mathrm{cm}}^{3}\right])$, $\;c_{t}$ denotes the number of aerosol particles per unit volume discharged during the conversation ($\left[\mathrm{Number}\;\mathrm{of}\;\mathrm{aerosol}\;\mathrm{particle}/{\mathrm{cm}}^{3}\right]),\;s$ denotes the ratio of speaking (0≤s<1), and b denotes the respiratory rate per unit time ($\left[\frac{{\mathrm{cm}}^{3}}{h}=\frac{1}{1000}\times \frac{l}{h}\right])$

#### 2.3.2. Distance Reduction Model

Sun and Zhai conducted a study on the effectiveness of social distancing and ventilation in preventing the spread of COVID-19 and presented a model for estimating the risk of exposure to droplets, based on distance from an infected person, as shown in Equation 5 [20]. Based on this model, a subject 1 m away from an infected person would be exposed to about 43% of the total discharged droplet mass, and a subject 2 m away would be exposed to about 30%.

(5) E(d)=−18.19ln(d)+43.276

E(d), with a value of 0–100%, denotes the risk of exposure to a droplet at a distance *d*.

This study aimed to establish a viral transmission model using the risk of exposure to droplets established by Sun and Zhai [20].

In the model showing the risk that subject B is infected by person A, *n*(B←A) represents the number of viruses discharged from infected person A to infect subject B. The number of viruses transferred is proportional to the number discharged and exposure time, as in the uniform distribution model. The number of viruses is assumed to decrease as the distance from the infected person increases. Therefore, *n2*(B←A) decreases with an increase in the distance between infected persons A and B. 

When infected person A and subject B are separated by a distance of *d* and stay together for *h* hours, subject B is exposed to the aerosol present in the space within the radius of the infected person. Because the number of viruses decreases with increasing distance, only *E(d)*% of the initial discharge of infected person A is delivered to the subject, and the number of discharged viruses can be expressed as in Equation (6):

(6) $$n2\left(B\leftarrow A\right)=\left(\frac{N}{f}\times h\right)\times \frac{E\left(d\right)}{100}\times \frac{b}{W\left(d\right))}\times \left(c\times v\right)\times \left(1-m\right)\times t$$

where N denotes the number of aerosol particles discharged by infected person A per unit time (Equation 4) used in the uniform distribution model, E(d) denotes the risk of exposure according to the distance, and *W*(*d*) denotes the volume of the space between the infected person and the subject ($\left[l=\frac{1}{1000}\times m^{3}\right])$. 

As the distance d between infected persons A and B increases, the volume *W*(*d*) of the space between the two people increases, and the number of transmitted viruses decreases by *W*(*d*)%. 

**Figure 2:**
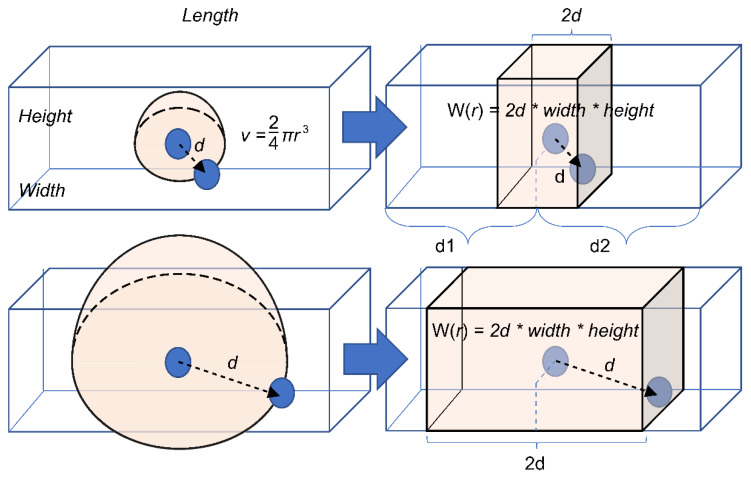
Simplified model for volume estimation of the space between two individuals in a vehicle.

If the aerosol discharged from the infected person spreads uniformly and three-dimensionally, it will spread in a spherical shape centered on the infected person. To simplify, a one-dimensional spread model is applied in this study, as shown in Figure 2. In other words, the volume of the space is proportional onlyto the straight-line distance between the two individuals by fixing the two axes of height and width. Because the aerosol discharged from the infected person spreads uniformly back and forth, the volume of space affecting the infected person can be calculated by simplifying it as in Equation (7).

(7) W(d)=1000×Width×Height×(Min(d1,d)+Min(d2,d))

### 2.4. Model for Calculating Infection Risk and the Number of Infected Individuals on Public Transportation

If there are *M* people in a public transportation vehicle, of which $m_{f}$ are infected, the risk of passenger *i* getting infected is calculated using Equation (8).

(8) $$P_{i}=\frac{M-m_{f}}{M}\sum_{\forall j|i\neq j}\frac{m_{f}}{M-1}P\left(i\leftarrow m_{j}\right)$$

The number of new infections caused by $m_{f}$ infected individuals in a public transportation vehicle with *M* passengers is calculated using Equation (9).

(9) $$N_{e}=\sum_{\forall i}P_{i}$$

The risk of one or more new infections caused by $m_{f}$ infected people in a public transportation vehicle is calculated using Equation (10).

(10) $$P\left(N_{e}\geq 1\right)=1-\prod_{\forall i}\left(1-P_{i}\right)$$

## 3. Results

### 3.1. Parameter Settings

#### 3.1.1. Probability of Infection by a Single Virus *p*

Lelieveld et al. calculated the probability of infection by a single virus using the infective dose (ID50) concept used in the medical community; it refers to the amount of virus required to infect 50% of the subjects [11]. 

If the probability of infection by a single virus is *p*, the probability of not being infected is (1 − *p*). Therefore, the probability of n individuals not being infected is ${\left(1-p\right)}^{n}$. If the *ID*50 concept is applied, Equation (8) indicates that the probability of not being infected by *ID*50 viruses is 0.5.

(11) $${\left(1-p\right)}^{ID50}=0.5\;$$

Equation (9) can be obtained by rearranging for *p*.

(12) $$p=1-0.5^{1/ID50}\;$$

Although there is no clear data about the *ID*50 value of COVID-19, Lelieveld et al. defined it as 316 based on information on similar viruses [11]. Substituting this into Equation 9, the probability of infection *p* by a single coronavirus becomes approximately 0.0022 (0.22%), which was the value used in this study.

#### 3.1.2. Probability of Deposition *t*

The probability of deposition, *t*, is the likelihood that the coronavirus reaches and deposits in the respiratory tract of the exposed person. As described above, not all viruses that a subject is exposed to are deposited, and only a portion reaches the respiratory tract. Lelieveld et al. stated that the probability of coronavirus deposition would be approximately 0.5 (50%) [11]; thus, it was set accordingly in our study.

#### 3.1.3. Number of Viruses per Unit Volume *c*

The specific amount and concentration of the virus in a person infected with COVID-19 have not yet been determined. Lelieveld et al. investigated these in the throat of an infected person under the assumption that respiratory droplets and aerosols discharged from an infected person contain the same number of viruses as the fluid in the respiratory tract that produces droplets and aerosols [11]. The number of viruses detected in the throat of an infected person was approximately $10^{4}\;\sim \;10^{11}$ per 1 mL; $5\times 10^{8}$ viruses per 1 mL were detected on average in those with symptoms of infection, accounting for one-fifth of those testing positive. Accordingly, the number of coronaviruses per unit volume was set to $5\times 10^{8}$ per 1 mL (${\mathrm{cm}}^{3})$. 

#### 3.1.4. Volume of Aerosol Particle *v*

To set the volume of the aerosol particles used in the model, the diameter must be known. Although the size of aerosol particles discharged through breathing and speaking varies, as mentioned above, most of the particles have a diameter of 1 μm. 

Meanwhile, small particles such as aerosols evaporate as they are discharged from the mouth. Lelieveld et al. assumed that the diameter was 5 μm but reduced to 1 μm due to evaporation while falling [11]. Therefore, in this study, the average volume of aerosol particles was set to $6.54\times 10^{-11}{\;\mathrm{cm}}^{3}$ under the assumption that the average volume of the aerosol particles was 5 μm.

#### 3.1.5. Number of Aerosol Particles per Unit Volume Discharged during Respiration and Conversation

Morawska et al. stated that the concentration of aerosol particles 0.3~20 μm generated during respiration and conversation is at the level of 0.1~1.1 ${\mathrm{cm}}^{-3}$ [21]. Lelieveld et al. assumed that 0.06 aerosol particles per 1 ${\mathrm{cm}}^{3}$ were discharged during respiration and 0.6 per 1 ${\mathrm{cm}}^{3}$ during conversation, considering that the effective average diameter of fine particles was 5 μm [11]. The same values were used in this study. 

#### 3.1.6. Respiratory Rate per Unit Time *b*

While the respiratory rate varies depending on individual characteristics, in adults it ranges from 6–8 L per minute. Accordingly, in this study, the respiratory rate per hour was set to 0.12 L, assuming that the rate per minute was 7 L.

### 3.2. Results of Analyzing Changes in the Risk of Infection on Public Transportation

The exposure time, ventilation rate, mask effectiveness, and conversation rate may vary depending on the operation and usage characteristics of the public transportation that is analyzed. These constitute the parameters in the model, and the risk of infection varies accordingly. To properly analyze the results of the scenario, it is necessary to first examine the changes in the risk of infection according to each parameter. Therefore, in the most basic scenario of a bus, the default parameters were set to 45 passengers, a conversation rate of 0.1, a ventilation rate of 3.3, mask effectiveness of 0.7, and an exposure time of 1 h. Changes in the number and risk of new infections were examined according to the changes in each parameter.

#### 3.2.1. Exposure Time

The risk of infection is proportional to the number of viruses discharged by an infected person, which is also proportional to the respiratory rate. Since the respiratory rate is proportional to the unit time, the longer the contact time with an infected person, the higher the risk of infection. The uniform distribution model was named Model 1, and the model of reduction with distance was named Model 2. With an exposure time of 1 h, the number of new infections was calculated as 0.0157 for Model 1 and 0.0156 for Model 2. This is interpreted as the probability of infecting 1.57 and 1.56 people, respectively, when an infected person traveling for 1 h uses a bus 100 times. In addition, the probability of one or more new infections in a vehicle is 1.56% for Model 1 and 1.55% for Model 2, which are interpreted as the probability of one or more new infections when an infected person uses the vehicle approximately 64 times.

**Figure 3:**
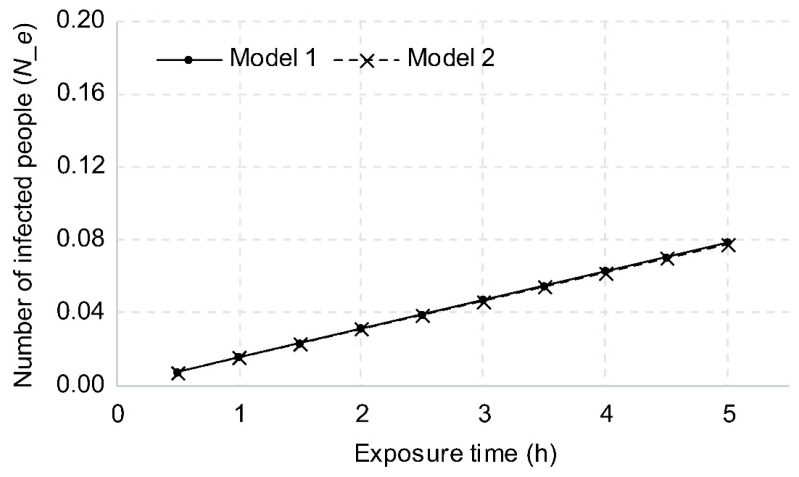
Changes in the number of new infections based on exposure time.

As shown in Figure 3, when the exposure time was extended from 1 h to 2 h, the probability doubled to 0.0315 and 0.0311, respectively, and when it was extended to 3 h, the risk was three times greater at 0.0472 and 0.467, respectively. The difference between Model 1 and Model 2 was insignificant, at approximately 1%, with the probability based on Model 1 being higher than that based on Model 2.

#### 3.2.2. Ventilation Rate

**Figure 4:**
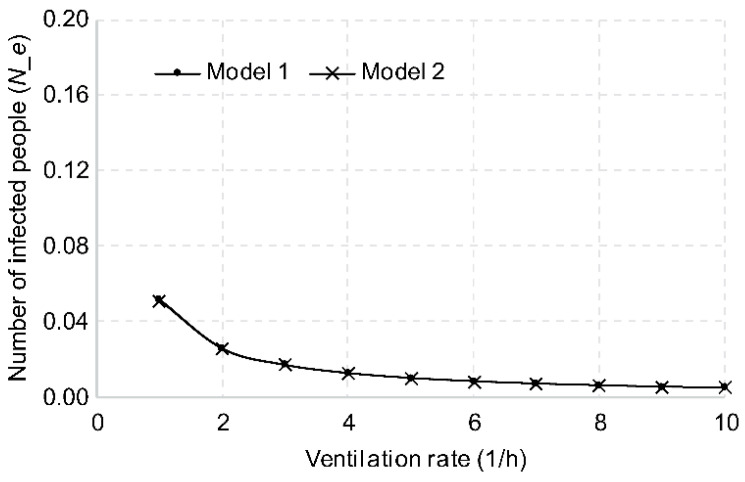
Changes in the number of new infections based on ventilation rate.

As mentioned above, ventilating the air inside a public transportation vehicle decreases the number of viruses discharged by an infected person as well as the risk of infection. In terms of changes in the risk of infection, when ventilating once per hour, the number of new infections was calculated as 0.0519 for Model 1 and 0.0514 for Model 2, as shown in Figure 4. When ventilation was increased to twice per hour, the number of new infections was reduced to 0.026 and 0.0257, respectively, and to 0.0173 and 0.0171, respectively, when ventilating three times per hour.

#### 3.2.3. Mask Effectiveness

**Figure 5:**
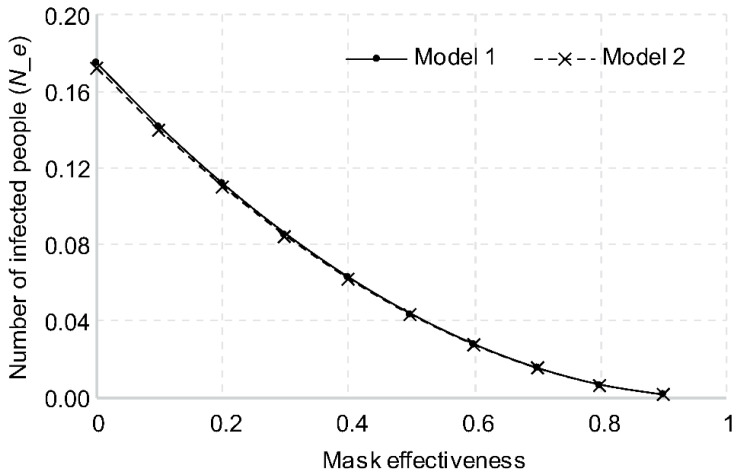
Changes in number of new infections based on mask effectiveness.

The higher the mask effectiveness, the lower the number of viruses discharged by an infected person. The number of viruses transferred to the respiratory tracts of the subjects would also decrease, thereby lowering the probability of infection. In terms of changes in the risk of infection, with mask effectiveness of 0 (not wearing a mask), the number of new infections was calculated as 0.1746 for Model 1 and 0.1723 for Model 2. KF-AD is known to block 55–80% of fine particles with an average size of 0.4–0.6 μm, as shown in Figure 5. If all passengers in a transportation vehicle are wearing a mask with KF-AD or higher grade, the average effectiveness is approximately 0.7. The number of new infections was calculated as 0.0157 for Model 1 and 0.0156 for Model 2, with the number of new infections reduced to 1/10 or less of the number when no masks were worn. 

#### 3.2.4. Frequency of Conversation

**Figure 6:**
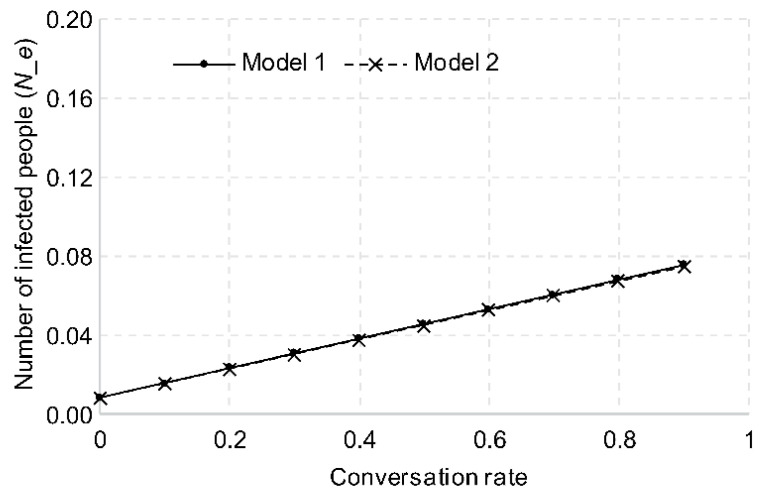
Changes in number of new infections based on conversation rate.

Since a higher number of viruses are released when conversing versus not, the more an infected person talks, the higher the risk of infection. When the infected person does not speak at all for 1 h, the aerosol is discharged only through respiration. The number of new infections, in that case, is calculated as 0.0083 for Model 1 and 0.0082 for Model 2, as shown in Figure 6. The number of new infections increased by approximately 0.9 times for each 10% increase in the conversation rate.

### 3.3. Analysis of Changes in the Risk of Infection on Public Transportation Due to Passenger Number Reduction

The greater the number of passengers in a vehicle, the higher the probability of the presence of an infected person and the risk of transmission. A policy to reduce the number of passengers in a vehicle may be considered to decrease the spread of COVID-19. 

Compared to short-distance travel of about 1 h, the probability of having a conversation with the person in the next seat or on the phone, as well as the probability of a mask falling off while talking or removing it because of stuffiness, increases during long-distance travel of about 3 h. In other words, it can be assumed that the conversation rate increases and the mask efficiency decreases during long-distance travel. In this study, the short-distance travel conditions were assumed to be a travel time of 1 h, conversation rate of 0.1, mask effectiveness of 0.7, and ventilation rate of 3.3, whereas long-distance travel conditions were 3 h, 0.2, 0.5, and 3.3, respectively. Changes in the risk of infection due to the reduction in the number of passengers were analyzed under short-distance and long-distance travel conditions. 

**Figure 7:**
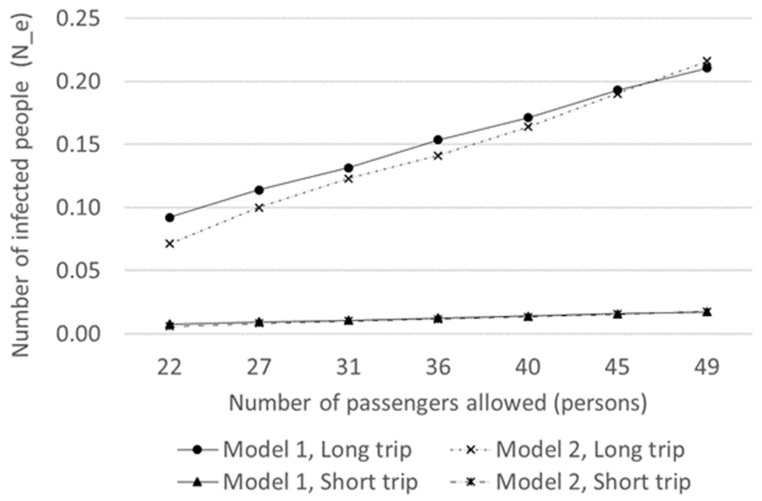
Changes in the risk of infection due to the reduction in the number of passengers under short- and long-distance travel conditions.

As shown in Table 2 and Figure 7, under short-distance travel conditions with 49 passengers in the vehicle, the number of new infections was 0.0172. When the number of passengers was reduced to 22, the number decreased from 0.00966 to 0.0075. In comparison, under long-distance travel conditions with 49 passengers, the number of new infections was 0.2105. When the number of passengers was reduced to 22, the number decreased from 0.11841 to 0.0921. Under both conditions, the reduction in the number of passengers and the risk of infection were analyzed. The number of new infections decreased more than 12 times under long- compared to short-distance travel. In other words, the reduction in the number of passengers was 12 times more effective in decreasing the risk of infection during long-distance travel.

### 3.4. Analysis of Changes in the Risk of Infection on Public Transportation Based on Distancing Strategy

Since the risk of COVID-19 infection depends on distance, it should also change based on the seating arrangement. We measured the difference in the risk of infection according to the seating arrangement by applying Model 2, assuming that virus transmission decreased with increasing distance. 

There must be empty seats in the arrangement. In this study, strategies were established for 22 seats on a bus that could accommodate a maximum of 45 passengers under short-distance travel conditions with a travel time of 1 h, conversation rate of 0.1, mask effectiveness of 0.7, and ventilation rate of 3.3. 

**Figure 8:**
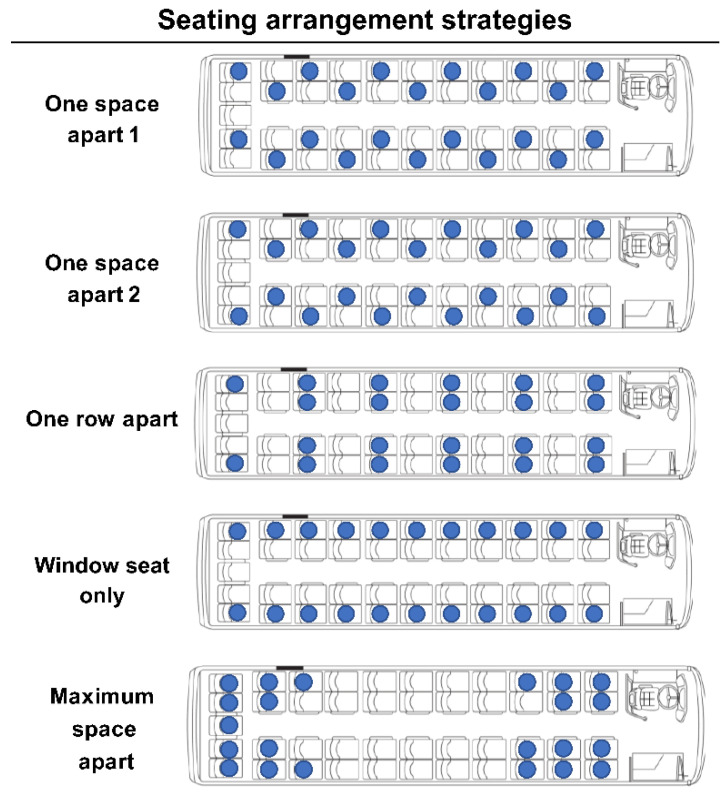
Seating arrangement strategies.

As shown in Figure 8, five arrangement strategies were set as comparative alternatives. The first was to arrange the seats one space apart in a zigzag, and the second was similar to the first but with the seats on both sides of the same row in a symmetrical fashion. The third strategy was to skip a row between the seats, and the fourth was to only use window seats. The last strategy was to maximize the total relative distance between 22 people by arranging the seats in the front and rear of the vehicle.

**Figure 9:**
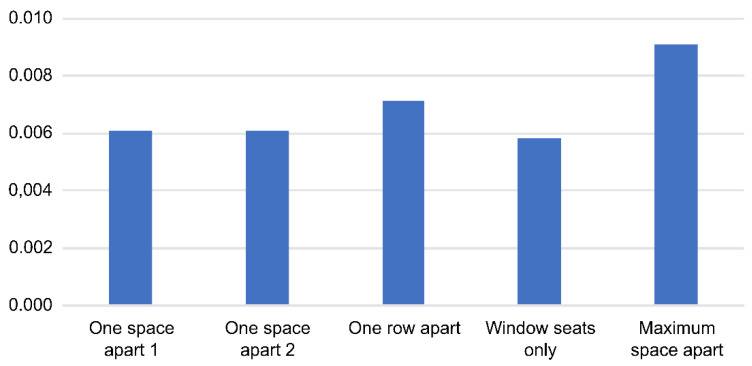
Number of new infections by seat arrangement strategy.

Table 3 and Figure 9 show the number and risk of new infections based on each of the five seating strategies. The risk was lowest for the window-seat-only strategy and highest for the maximum space apart. Separating all passengers as far apart as possible from each other seems to be the best to minimize the risk of infection. 

## 4. Discussion

COVID-19 is spread by droplets and aerosols discharged through the human respiratory tract; the probability of contracting it is proportional to the number of viruses a subject is exposed to. The higher the respiratory rate of an infected person, the higher the risk. Therefore, the risk of infection in a public transportation vehicle increases in proportion to the exposure time with an infected person, implying higher risk for long-distance travel. The risk is also higher for intercity compared to city buses, inter-regional compared to intercity buses, and the Korea Train eXpress(KTX) compared to the metro.

More droplets and aerosols are discharged during conversation compared to respiration, which leads to a higher number of discharged viruses and an increased risk of infection on public transportation. The risk is doubled at a conversation rate of 10% and tripled at 20%. Therefore, when using public transportation, it is necessary to minimize phone calls and verbal communication with other passengers.

Most of the droplets are blocked by masks due to their large particle size, but since aerosols are smaller, some leaks can occur. The higher the mask effectiveness, the more viruses are filtered, and the more the transmission to the respiratory tract of the subject is reduced, resulting in an overall infection risk reduction on public transportation. As previous studies indicated [22,23], masks are undoubtedly crucial, and wearing any type is better than not wearing one at all. A KF-AD mask that blocks droplets reduces the risk of infection by 10 times when compared to not wearing a mask, and a KF94 or a higher-grade mask reduces the risk by 100 times. 

Aerosols are small particles that float in the air instead of droplets that fall immediately. Therefore, the number of viruses in the air can increase over time in unventilated indoor spaces. When there is good ventilation, indoor air is replaced by outdoor air, decreasing the number of viruses in the room and decreasing the risk of infection. When ventilation is performed twice per hour, the risk of infection is reduced by half, and when it is performed three times per hour, it is reduced to a third. Unlike city buses and metros, where ventilation occurs naturally while stopping every 2–3 min to open and close the vehicle doors, natural ventilation is likely to occur less frequently on intercity buses that make fewer stops. Inter-regional buses traveling between regions are even less likely to be ventilated. An air conditioner may be considered as an in-vehicle ventilation system, but circulating the air inside without a virus filter will only spread it more, which does not help to lower the risk of infection. Therefore, a ventilation system equipped with a virus filter is needed to reduce the risk of infection on public transportation.

Long-distance travel is associated with longer in-vehicle travel time, which is likely to increase the probability of talking and taking off one’s mask. These factors significantly increase the risk of infection. To counteract this, the number of passengers on public transportation must be limited; this can reduce the risk of infection by 12 times for long-distance travel. 

Since aerosols float in the air, the risk of infection may be perceived as equal for everyone within that space. However, those close to an infected person are more likely to be affected than those far away. If the risk of infection varies according to distance from the infected person, it should vary according to the seat arrangement in the vehicle as well. We found that the window-seat-only strategy lowered the risk of infection the most, while the maximum-space-apart strategy exhibited the highest risk of infection. Compared to the maximum-space-apart strategy, the window-seat-only strategy showed a 0.0033 lower number of new infections and a 0.328% lower risk. This is similar to the effect of reducing the number of passengers by 20% of the maximum capacity or by 9–10 people for short-distance travel. When the number of passengers per vehicle approaches the maximum capacity due to high traffic demand, it is necessary to consider a plan to reduce the number of passengers in order to improve the service level by reducing congestion in the vehicle while lowering the probability of infection. If not, applying the strategy of spacing passengers as far apart as possible is expected to have a similar effect.

In this study, the spread of COVID-19 was modeled considering exposure time, mask efficiency, the ratio of speaking, and ventilation rate. Using the model, the probability of and the number of new infections were estimated according to the number of viruses spreading in public transportation vehicles. Since there are no significant statistical data on the probability of COVID-19 infection from public transportation use, and such a study cannot be conducted in real life, we could not verify the accuracy of the results in this study.

It is difficult to directly compare the results of previous studies because of the different assumptions and conditions. Under the conditions of 1 h travel time, conversation rate of 0.1, mask effectiveness of 0.7, and ventilation rate of 3.3, the infection risk in this study was 1.56% in the uniform distribution and 1.55% in the distance reduction model. The RSSB results were 0.2% [10], which is much lower than our study because they assumed a very low probability of contact with an infected person. Lelieveld et al. had a result of 1.60% [11], which is almost the same result as ours, and their model had the same structure as the uniform distribution model in our study. 

As noted to date, the results may vary depending on the assumptions of the analysis. Therefore, rather than the absolute values of the probability of infection and the number of infected individuals, it is more important to decide on policies by analyzing variations according to each variable and scenario.

## 5. Conclusions

Public transportation is essential to guarantee mobility in daily life. Nevertheless, people worldwide are reducing travel and avoiding public transportation due to COVID-19 [23,24,25,26]. Since public transportation appears to play an important role in the spread of COVID-19, it faces a major crisis. Therefore, more measures are needed to prevent the spread of COVID-19 on public transportation [27,28,29,30]. As Przybylowske et al. suggested, to prevent people from avoiding public transportation due to the risk of COVID-19 infection, the government should provide accurate guidelines and implement appropriate policies to help people perceive public transportation as safe [25]. 

Wang et al. emphasized that precautionary measures, such as assessing ventilation, airflows, air filtration, UV disinfection, and mask fit, must be implemented to mitigate aerosol transmission at both short and long ranges [19]. In this study, two infection risk estimation models were proposed to examine the correlation between these various factors and the infection risk in public transportation through sensitivity analysis of major factors. The analysis showed that the risk of infection in public transportation vehicles can be minimized through mask-wearing, prohibiting food intake, limiting conversations, and ventilation, similar to the findings noted in previous studies. Among these factors, mask-wearing and ventilation were the most important factors. In addition, reducing the number of passengers and distributing the seating arrangement were found to significantly reduce the risk of infection in public transportation. 

However, the proposed models have a limitation, in that they do not reflect all external environmental factors, such as temperature. Therefore, future studies should focus on developing new models (considering, for example, the effects of temperature) and modifying existing ones to include the factors addressed in this study. In addition, the models developed in this study can be used for evaluating the effectiveness of measures to reduce the number of passengers and distribute the seating arrangement; it may also be used for infectious diseases other than COVID-19.

## Figures and Tables

**Table 1:** KF mask certification grades and standards.

Grade	Dust Collection Efficiency	Inhalation Resistance	Inward Leakage
KF80	80% or higher (Sodium chloride test)	60 Pa or lower	25.0% or lower
KF94 *	94% or higher (Sodium chloride and paraffin oil tests)	70 Pa or lower	11.0% or lower
KF99	99% or higher (Sodium chloride and paraffin oil tests)	100 Pa or lower	5.0% or lower

Source: Ministry of Food and Drug Safety (2021), Guidelines for Standard Specifications for Filtering Respirators. * KF94 masks certified by South Korea show similar performance to N95 masks certified by the US.

**Table 2:** Analysis of the risk of infection with passenger number reduction under short- and long-distance travel conditions.

Number of Passengers	Short-Distance Travel				Long-Distance Travel			
	New Infections (People)		Risk of New Infections		New Infections (People)		Risk of New Infections	
	Model 1	Model 2	Model 1	Model 2	Model 1	Model 2	Model 1	Model 2
22	0.0075	0.0058	0.75%	0.58%	0.0921	0.0713	8.82%	6.89%
27	0.0093	0.0082	0.93%	0.81%	0.1140	0.0999	10.80%	9.53%
31	0.0107	0.0101	1.07%	1.00%	0.1316	0.1231	12.35%	11.60%
36	0.0125	0.0115	1.24%	1.15%	0.1535	0.1411	14.26%	13.18%
40	0.0140	0.0134	1.39%	1.33%	0.1710	0.1637	15.75%	15.13%
45	0.0157	0.0156	1.56%	1.55%	0.1930	0.1903	17.58%	17.36%
49	0.0172	0.0177	1.70%	1.75%	0.2105	0.2159	19.02%	19.46%

**Table 3:** Number and risk of new infections by seat arrangement strategy.

Seat Arrangement Strategy	Number of New Infections (People)	Risk of New Infections (%)
One space apart 1	0.00610	0.609%
One space apart 2	0.00612	0.610%
One row apart	0.00712	0.709%
Window seat only	0.00583	0.581%
Maximum space apart	0.00913	0.909%

## Data Availability

No new data were created or analyzed in this study. Data sharing is not applicable to this article.
